# The Programme of the Hospital Saturday Fund

**Published:** 1891-07-18

**Authors:** Reginald B. D. Acland

**Affiliations:** Chairman of the Fund


### THE PROGRAMME OF THE HOSPITAL SATURDAY FUND.

By Mr. REGINALD B. D. ACLAND, Chairman of the Fund.

I HAVE no intention of being drawn into a controversy on the aims and objects of the Hospital Saturday Fund with an anonymous writer professedly so omniscient, practically so very inaccurate as "Agricola." My grievance was, and still is, that while professing to comment on my evidence before the House of Lords' Committee, he either gives the go-by to, or distorts what I actually did say; of which unfortunate proclivity—as I suppose I must consider it—he gives another instance in the last paragraph but one of his last communication.

Just a few words, however, by your permission, on the general question. I entirely agree that the letter system is "logically indefensible," and I should be glad to see it abolished; but it seems to me extremely unfair that the authorities of the Hospital Saturday Fund—which is composed of the small contributions of very large numbers of working people—should be singled out for attack when in accepting, or receiving, or asking for (whichever you like) letters of recommendation they are only doing exactly what is done by individual subscribers and by every other similar body, including the Hospital Sunday Fund, which in many, if not all, cases, receive letters of recommendation on a scale similar to that which is applied to the Saturday Fund.

The argument based on "the probability equivalent to a certainty that every letter will . . . be made to do duty"—which, like most of "Agricola's" statements or suggestions about the Saturday Fund, is quite inaccurate—entirely fails, unless two facts can be proved: first, that any increase in the number of patients at the hospitals receiving a grant from the Hospital Saturday Fund is directly due to the operations of that body; that is to say, unless it can be proved that the great bulk of the patients going with letters of recommendation from the Saturday Fund would not have found their way to the hospitals with letters received from ordinary subscribers; and second, that the amount contributed by the Saturday Fund to hospitals as a whole is insufficient to pay the cost of the patients sent by that fund to the hospitals. Neither of these facts ever has been, nor, I believe, can be proved. Even if either or both of these propositions were established, it would still have to be considered, whether the proper way of looking at the contributions of the Saturday Fund would not be as a contribution by patients or their friends towards their cost, the fund being in the main contributed by those who most people consider to be proper persons to be treated at the hospitals.

In conclusion, I may perhaps be allowed to add this. I suppose that the managers of the different hospitals know their own interests at least as well as "Agricola." Yet, notwithstanding the fact—if it be a fact—alleged by "Agricola" that the Fund "will require, for every guinea contributed, several guineas' worth of relief for its nominees," twenty-seven general and fifty-nine special hospitals, besides numbers of dispensaries, convalescent homes, and other medical charities apply year after year for a grant from the Fund. I believe some one has said that three is the least number which can be "several," and taking "Agricola's" "several" at that figure, it is appalling to think that the £132,000 contributed in seventeen years by the Hospital Saturday Fund to the Metropolitan medical charities has cost those charities considerably over a quarter of a million of money in addition to the amount received. No wonder we are told that the hospitals are approaching insolvency!

THE *Times* correspondent, writing from St. Petersburg, mentions a curious case of superstition which has been the subject of a trial before the Criminal Sessions Court at Samara. Six peasants were sentenced for disinterring the body of a woman who had died from drink, and floating it down the Volga. Their object in doing this arose from a fixed belief among the Russian peasantry that if the dead body of a drunkard is thrown into a river rain will ensue. This is a novel and unpleasant manner of combating a drought, and unfortunately in Russia there is no lack of the required medium for perpetuating such an unsavoury superstition.

